# Spirobenzofuran Mitigates Ochratoxin A-Mediated Intestinal Adverse Effects in Pigs through Regulation of Beta Defensin 1

**DOI:** 10.3390/toxics12070487

**Published:** 2024-07-03

**Authors:** Jung Woong Yoon, Myoung Ok Kim, Sangsu Shin, Woo-Sung Kwon, Soo Hyun Kim, Yun-Ju Kwon, Sang In Lee

**Affiliations:** 1 Department of Animal Science and Biotechnology, Kyungpook National University, Sangju 37224, Gyeongsangbuk-do, Republic of Korea; kizx789@knu.ac.kr (J.W.Y.); ok4325@knu.ac.kr (M.O.K.); sss@knu.ac.kr (S.S.); wskwon@knu.ac.kr (W.-S.K.); 2 Research Institute for Innovative Animal Science, Kyungpook National University, Sangju 37224, Gyeongsangbuk-do, Republic of Korea; 3 National Institute for Korean Medicine Development, Gyeongsan 38540, North Gyeongsang, Republic of Korea; beluga81@nikom.or.kr (S.H.K.); mars005@nikom.or.kr (Y.-J.K.)

**Keywords:** Ochratoxin A, IPEC-J2 cells, DEFB1 overexpression cell line, natural products, high-throughput screening, Spirobenzofuran

## Abstract

Antimicrobial peptides (AMPs) function to extensively suppress various problematic factors and are considered a new alternative for improving livestock health and enhancing immunomodulation. In this study, we explored whether AMP regulation has positive influences on Ochratoxin A (OTA) exposure using a porcine intestinal epithelial cell line (IPEC-J2 cells). We constructed a beta-defensin 1 (DEFB1) expression vector and used it to transfection IPEC-J2 cells to construct AMP overexpression cell lines. The results showed that OTA induced cytotoxicity, decreased cell migration, and increased inflammatory markers mRNA in IPEC-J2 cells. In DEFB1 overexpressing cell lines, OTA-induced reduced cell migration and increased inflammatory markers mRNA were alleviated. Additionally, a natural product capable of inducing DEFB1 expression, which was selected through high-throughput screening, showed significant alleviation of cytotoxicity, cell migration, and inflammatory markers compared to OTA-treated IPEC-J2 cells. Our finding provides novel insights and clues for the porcine industry, which is affected by OTA exposure.

## 1. Introduction

Ochratoxin A (OTA), a toxic metabolite derived from *Aspergillus* and *Penicillium* molds, is a problematic mycotoxin that can contaminate various raw materials used in processed food and feed products, including cereals, vegetables, nuts, fruits, and forage [1,2]. Consequently, OTA poses a significant threat to all food systems. Animal feed, in particular, is becoming increasingly susceptible to OTA contamination compared to clean and safe human food, leading to detrimental diseases, such as bleeding, sepsis, dysfunction, lesions, and toxicity in various organs. The resulting organ damage often translates to reduced growth rates and, ultimately, economic losses in the livestock industry [3,4,5]. Notably, OTA is readily absorbed in the gastrointestinal tract, particularly within the jejunum, with several studies highlighting its ability to cause intestinal damage in vivo and in vitro by inducing malfunction and through various molecular mechanisms [6,7]. Although various effects and molecular mechanisms have been demonstrated, effective solutions and alternatives remain critical due to the gradual increase in OTA-induced problems.

The intestinal epithelium, covered with a single layer of connected epithelial cells, serves as a critical interface facilitating interactions between the host and its internal and external environments [8]. Intestinal epithelial cells maintain homeostasis by facilitating nutrient absorption while impeding the invasion of harmful factors, such as pathogens, bacteria, natural toxins, and contaminated food [9]. However, if the intestinal epithelium accumulates damage from external factors, intestinal barrier integrity will be compromised, resulting in the transmission of harmful factors across the intestinal wall and, ultimately, immune responses [10]. To maintain intestinal integrity, immunocytes trigger the production of inflammatory cytokines, chemokines, leukocytes, immunoglobulins, and antimicrobial peptides (AMPs) through various mechanisms [11,12,13]. However, overactivation of these elements induces adverse effects, such as inflammation, weight loss, fever, fatigue, and digestive issues, rendering the host vulnerable [14,15]. Consequently, consuming sanitary feed and maintaining proper immunity is necessary to preserve intestinal integrity within the livestock industry. Nonetheless, livestock remain susceptible to OTA exposure, as the consumption of OTA-contaminated feed is often inevitable. Moreover, previous studies have shown that OTA exposure damages the intestinal epithelium and activates immune responses through various signaling pathways [16,17,18].

AMPs, which are part of an organism’s innate immune system, comprise small peptides and are produced in various cells, including skin, epithelial, and immune cells [19]. AMPs not only eradicate pathogenic bacteria, viruses, and fungi through direct antimicrobial activity but also play an extensive role in proliferation, wound healing, immune system regulation, and inflammatory cytokine inhibition [20,21,22]. Several studies have suggested that the exogenous supplementation or endogenous synthesis of AMPs affects animal health and productivity by regulating intestinal integrity and homeostasis [23,24,25]. Recently, defensins, one of the AMP subclasses, were reported to be mainly secreted by the epithelium and have been shown to positively influence adverse conditions in various intestinal epithelial models [26,27]. Thus, we hypothesized that the overexpression of beta-defensin could serve as an effective intervention to mitigate OTA-induced complications in the intestinal epithelium.

Natural products, which are derived from living organisms in nature, offer various benefits, such as improved digestion and mineral binding, as well as antioxidant, anticancer, anti-inflammatory, and antibacterial activities [28]. Several studies have revealed that natural products improve health and mitigate risks associated with inflammation, cancer, diabetes, and various organ-related diseases, rendering them suitable as feed additives for livestock [29,30]. In this study, we aimed to explore AMP elevation as a potential alternative for protecting the intestinal epithelium against OTA exposure. Consequently, we screened 288 natural products and selected those that induce AMP, suggesting them as potential alternatives for protecting an OTA-exposed intestinal epithelium.

In this study, we ascertained whether the exogenous supplementation or regulation of endogenous synthesis of beta-defensin 1 (DEFB1) could serve as a viable alternative to address OTA-mediated issues in porcine small intestinal epithelial cells.

## 2. Materials and Methods

### 2.1. Culture and Treatment in IPEC-J2 Cells

IPEC-J2 cells, derived from the jejunal epithelium of piglets, were cultured in a mixture consisting of 90% Dulbecco’s Modified Eagle Medium (DMEM), supplemented with 10% fetal bovine serum and 1% penicillin–streptomycin from Thermo Fisher Scientific (Waltham, MA, USA). The cells were incubated at 37 °C with a 5% CO_2_ concentration. OTA was purchased from Sigma-Aldrich (St. Louis, MO, USA) and diluted in dimethyl sulfoxide (DMSO). For cell treatment, OTA solution was diluted in DMEM, with OTA concentration set at 5.5 µM based on our previous study [31].

### 2.2. Cell Viability

Cells were seeded in 96-well plates at 1 × 10^4^/100 µL per well and incubated for 33 h. Afterward, the cells were incubated in DMEM overnight, followed by treatments to each well. The cells were incubated at various durations (0 h, 24 h, 48 h, and 72 h), and 10 µL of EZ-Cytox (DoGenBio, Seoul, Republic of Korea) was added to each well. The reaction was incubated for two hours, and dye absorbance was measured using a GloMax Discover Multi-Microplate Reader (Promega, Madison, WI, USA) by subtracting the background reading at 600 nm from that at 450 nm.

### 2.3. Scratch Assay

Cells were seeded in migration cell culture dishes (SPL, Pocheon-si, Republic of Korea) at a concentration of 2–3 × 10^4^/100 µL and cultured until a cell monolayer was formed. Next, the cells were cultured in DMEM for 6–12 h. Subsequently, treatments were added, the culture inserted was removed, and cell movement was recorded at 6 h intervals using an inverted microscope (Korealabtech, Seongnam-si, Republic of Korea).

### 2.4. Quantitative Real-Time Polymerase Chain Reaction (RT-qPCR)

RNA extraction was conducted using The AccuPreP Universal RNA extraction kit (Bioneer, Daejeon, Republic of Republic of Korea), while Total RNA was quantified using a P200 Micro-volume spectrophotometer (Biosis Design, Gwangmyeong-si, Republic of Korea). For cDNA synthesis, reverse transcription of RNA (1 µg) was conducted using the DiaStar RT Kit (SolGent, Daejeon, Republic of Korea). Primers for amplifying the target gene used in RT-qPCR were designed using the Primer 3 program (https://primer3.ut.ee). RT-qPCR was conducted under specific reaction conditions using a 7500 Fast Real-Time PCR system: initial denaturation at 95 °C for 3 min, followed by 39 cycles of denaturation at 95 °C for 15 s, annealing at 56–60 °C for 15 s, and elongation at 72 °C for 15 s. Glyceraldehyde-3-phosphate dehydrogenase (GAPDH), a housekeeping gene, was used as a control for normalizing mRNA expression levels. Relative mRNA expression was calculated using the 2^−∆∆CT^ method: ∆Ct = Cq (treated) − Cq (control), and ∆∆Ct = ∆Ct (treated) − ∆Ct (control). The primer sequences used in this study are specified in Table 1.

### 2.5. Vector Construction

We designed the porcine DEFB1 gene using the Gene Synthesis service (Bionics, Seoul, Republic of Korea). The DEFB1 vector consisted of Backbone (pUC57-Kana) and Insert (DEFB1 coding sequence). Enzyme cutting was conducted on DEFB1 and pcDNA3.1(+) vector using BamH1 and Not1 (New England Biolabs, Ipswich, MA, USA). Each DNA was separated using 1% agarose gel electrophoresis at 100 V for 30 min, and DNA fragments were extracted using the AccuPreP PCR/Gel Purification Kit (Bioneer). Using T4 DNA Ligase (Bioneer), ligate Backbone DNA fragment (pcDNA3.1(+)), and Insert DNA fragment (DEFB1) to construct the pcBD1 vector.

### 2.6. Cell Selection

IPEC-J2 cells were seeded in a 6-well plate at 2 × 10^5^/3 mL per well and cultured until 70–80% confluence was achieved. We mixed the enzyme-cut pcBD1 DNA using BglII (NEB) and Lipofectamine 3000 Transfection Reagent (Invitrogen, Carlsbad, CA, USA). Opti-MEM medium was added to the mixture and incubated for 15 min. Afterward, the DNA–Lipofectamine complex was extensively applied to IPEC-J2 cells. Transfected cells were incubated for 48 h and grown in G418 sulfate (1000 ng/mL, Biosesang, Yongin-si, Republic of Korea) diluted in DMEM for 10–14 d. The cells obtained through this method are named pcBD1, a DEFB1 overexpression cell line.

### 2.7. HTS Assay and Validation for the Discovery of AMP-Induced Natural Products

For the HTS assay, 288 natural products were provided by the National Institute for Korean Medicine Development (Gyeongsan, Republic of Korea). All products were diluted to 1 mg/mL using DMSO. IPEC-J2 cells were grown at 1 × 10^4^/100 µL in 96 wells for 33 h and incubated with DMEM overnight. The cells were treated with a mixture of OTA (5.5 μM) and natural products at a final concentration of 20 ng/µL for 24 h. After treatment, 10 µL of EZ-Cytox was added to each mixture, and the reaction was allowed to proceed for 2 h. To evaluate absorbance between reactions, dye absorbance was measured by subtracting the background reading at 600 nm from that at 450 nm using a GloMax Discover Multi-Microplate Reader.

To validate candidate natural products, IPEC-J2 cells were seeded in 96-well plates under the above culture conditions. The cells were treated with various concentrations (2.5 ng/μL, 5 ng/μL, 10 ng/μL, 20 ng/μL, and 40 ng/μL) of each product. The dye absorbance at each concentration was compared with the control to select the optimal concentration for subsequent experiments. Subsequently, RNA extraction, cDNA synthesis, and RT-qPCR were conducted on each product under the conditions used in RT-qPCR to measure the mRNA expression of DEFB1.

### 2.8. Statistical Analysis

All experiments were independently conducted three times. The data were analyzed for significant differences between control and treatments using the general linear model (PROC-GLM) procedure in SAS. Graphed data were represented by means and standard errors. Comparison of statistical data was analyzed using a *t*-test and GLM. A *p*-value below 0.05 was considered statistically significant. Duncan’s multiple range test was used to evaluate the significant differences between the treatment groups.

## 3. Results

### 3.1. Effects of Ochratoxin A Treatment on Cytotoxicity, Migration, and Inflammatory Markers in IPEC-J2 Cells

**Figure 1:**
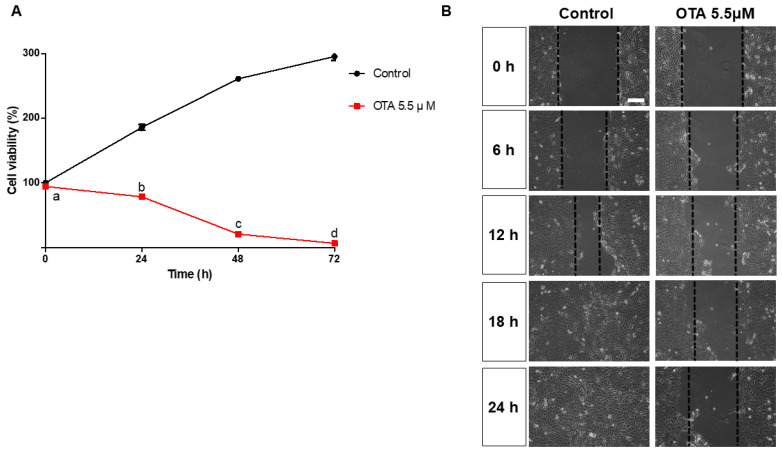
Ochratoxin A affects cytotoxicity and cell migration in IPEC-J2 cells. (**A**) Analysis of cell viability following OTA 5.5 μM treatment in IPEC-J2 cells compared with the control group at 0, 24, 48, and 72 h. (**B**) Effect on cell migration in IPEC-J2 cells after OTA 5.5 μM treatment at 0, 6, 12, 18, and 24 h. Lowercase letters (a, b, c, d) denote statistical differences between the control and the treatment groups. Error bars represent the standard errors (SEs) of three independent experiments. Scale bar = 40 μm.

To assess OTA cytotoxicity, IPEC-J2 cells were treated with OTA 5.5 μM in a time-dependent manner (0 h, 24 h, 48 h, 72 h). The results show a significant difference between the control groups and OTA treatment groups, as well as significant decreases in IPEC-J2 cells in a time-dependent manner (24 h, 48 h, 72 h) (Figure 1A). Subsequently, scratch assays were performed after a 24 h OTA 5.5 μM treatment in IPEC-J2 cells, revealing significant time-dependent differences in wound width compared to the control groups (Figure 1B).

**Figure 2:**
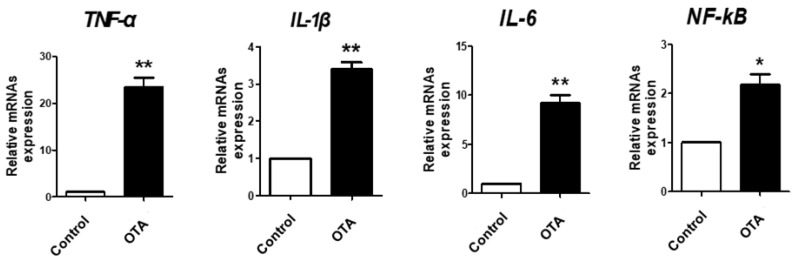
Ochratoxin A activates the expression level of inflammatory markers in IPEC-J2 cells. The relative mRNA levels of inflammatory markers (*TNF-α*, *IL-1β*, *IL-6*, and *NF-kB*) after OTA 5.5 μM treatment compared with the control group. * *p* < 0.05 and ** *p* < 0.01 denote statistical differences between the control and the treatment groups. Error bars represent the standard errors (SEs) of three independent experiments.

We then evaluated the mRNA levels of several inflammatory markers, such as *TNF-α*, *IL-1β*, *IL-6*, and *NF-kB*, using RT-qPCR to determine the effect of OTA treatment on inflammation in IPEC-J2 cells. The results showed that OTA treatment significantly increased mRNA levels of *TNF-α*, *IL-1β*, *IL-6*, and *NF-kB* compared to the control cells (Figure 2).

### 3.2. Alleviative Effect of Ochratoxin A Exposure in pcBD1 Cells

**Figure 3:**
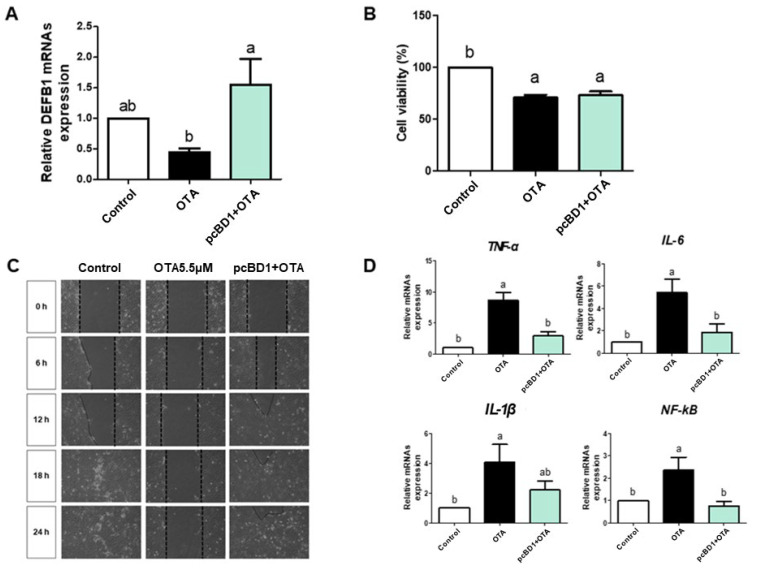
Ochratoxin A reduces the expression level of DEFB1, and the effect of DEFB1 increases cytotoxicity, cell migration, and inflammatory markers. (**A**) Relative mRNA levels of DEFB1 following OTA 5.5 μM treatment in IPEC-J2 cells and pcBD1 cells compared with the control group. (**B**) Analysis of cell viability following OTA 5.5 μM treatment in IPEC-J2 cells and pcBD1 cells compared with the control group. (**C**) Effects of OTA treatment on cell migration in IPEC-J2 cells and pcBD1 cells at 0, 6, 12, 18, and 24 h. (**D**) The relative mRNA levels of inflammatory markers (*TNF-α*, *IL-1β*, *IL-6*, and *NF-kB*) following OTA 5.5 μM treatment in IPEC-J2 and pcBD1 cells compared with the control group. Lowercase letters (a, b) denote statistical differences between the control and the treatment groups. Error bars represent the standard errors (SEs) of three independent experiments.

We used pcBD1, a DEFB1 overexpression cell line, to determine whether increasing DEFB1 affects OTA exposure. The mRNA expression of DEFB1 in OTA-treated IPEC-J2 cells was significantly decreased compared to the untreated group, while the mRNA expression of DEFB1 was significantly increased in OTA-treated pcBD1 cells compared to OTA-treated IPEC-J2 cells (Figure 3A). Cytotoxicity results showed that both IPEC-J2 and pcBD1 cells treated with OTA exhibited significant reductions in cell viability compared to the control (Figure 3B). Upon OTA exposure, the wound width of IPEC-J2 cells increased, while that of pcBD1 cells reduced (Figure 3C). The mRNA expression of inflammatory markers (*TNF-α*, *IL-1β*, *IL-6*, and *NF-kB*) in OTA-treated pcBD1 cells was significantly reduced compared to OTA-treated IPEC-J2 cells (Figure 3D).

### 3.3. High-Throughput Screening for Identifying Candidate Natural Products against Ochratoxin A Toxicity in IPEC-J2 Cells

**Figure 4:**
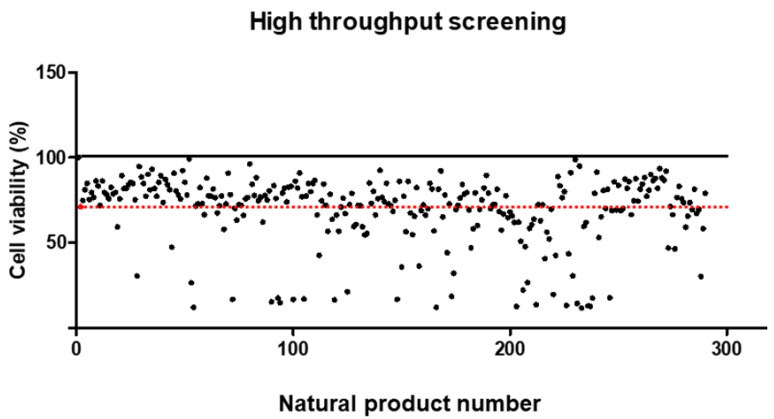
Effect of individual natural products on Ochratoxin A toxicity. IPEC-J2 cells were treated with OTA 5.5 μM and 20 ng/μL of each natural product for 24 h. Dots in the graph represent the control group, OTA group, and cotreatment group (OTA and 288 natural products), while the dotted line describes the cell viability value of the OTA group.

To identify candidate natural products with mitigating effects on OTA toxicity, we screened 288 natural products, along with OTA, in IPEC-J2 cells. Interestingly, 177 natural products exhibited higher cell viability compared to individual OTA treatments (Figure 4). Notably, we identified 17 natural products with cell viability exceeding 90% as potential candidates for mitigating OTA toxicity.

### 3.4. Cytotoxicity Validation of Candidate Natural Products in IPEC-J2 Cells

**Figure 5:**
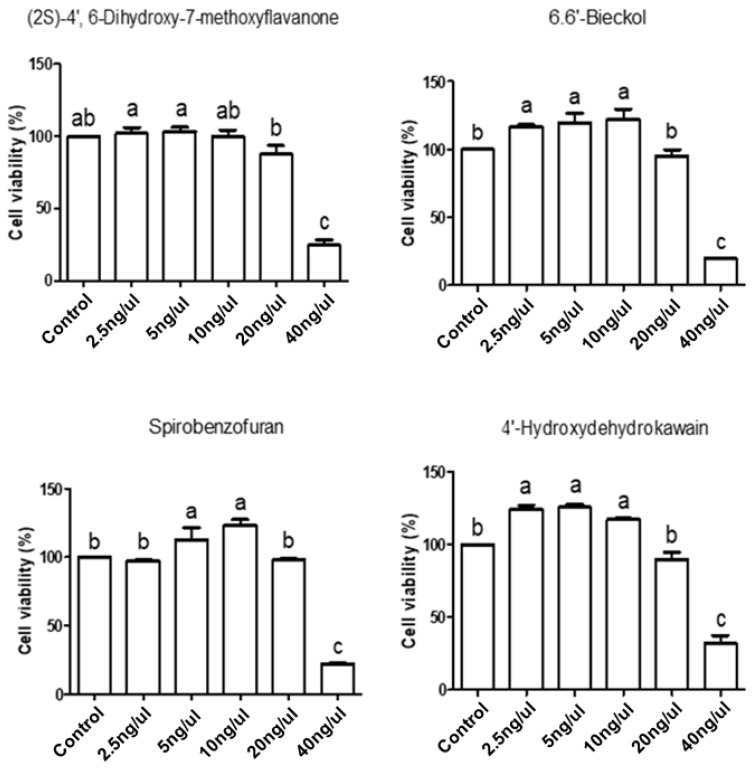
Analysis of cell viability using four candidate natural products in IPEC-J2 cells. IPEC-J2 cells were treated with various concentrations (2.5 ng/μL, 5 ng/μL, 10 ng/μL, 20 ng/μL, and 40 ng/μL) of (2S)-4′,6-Dihydroxy-7-methoxyflavanone, 6,6′-Bieckol, Spirobenzofuran, and 4′-Hydroxydehydrokawain for 24 h. Lowercase letters (a, b, c) denote statistical differences between the control and the treatment groups. Error bars represent the standard errors (SEs) of three independent experiments.

To determine the optimal concentration of candidate natural products in IPEC-J2 cells, cell viability assays were conducted at various concentrations (0 ng/μL, 2.5 ng/μL, 5 ng/μL, 10 ng/μL, 20 ng/μL, and 40 ng/μL) of (2S)-4′,6-Dihydroxy-7-methoxyflavanone, 6,6′-Bieckol, Spirobenzofuran, and 4′-Hydroxydehydrokawain for 24 h. When administered at a concentration of 10 ng/μL to IPEC-J2 cells, these natural products exhibited cell viability that was the same as that of the control or even significantly increased (Figure 5). Based on these results, we set the concentration of natural products to 10 ng/μL for 24 h in subsequent experiments.

### 3.5. DEFB1-Inducing Natural Products in IPEC-J2 Cells

**Figure 6:**
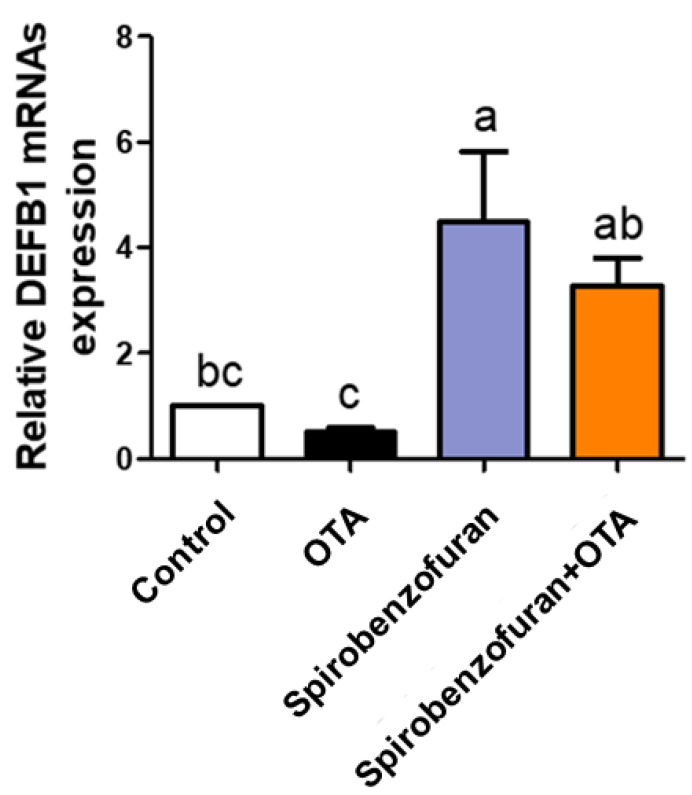
The candidate natural product improves the inhibition of DEFB1 caused by Ochratoxin A. The relative mRNA levels of DEFB1 following OTA 5.5 μM, Spirobenzofuran 10 ng/μL, and cotreatment (OTA 5.5 μM and Spirobenzofuran 10 ng/μL) in IPEC-J2 cells. Lowercase letters (a, b, c) indicate statistical differences between the control and the treatment groups. Error bars represent the standard errors (SEs) of three independent experiments.

To identify the natural products capable of inducing DEFB1 expression, we measured the mRNA levels of *DEFB1* for the four candidate natural products in IPEC-J2 cells, with or without OTA treatment, using RT-qPCR. The results showed significant increases in DEFB1 mRNA expression levels in the Spirobenzofuran treatment groups, whereas the other three natural products exhibited abnormal increases (Figure 6 and Supplemental Figure S1). These results speculated that Spirobenzofuran was the selected natural product for mitigation of OTA toxicity.

### 3.6. Regulatory Effects of the Selected Natural Products on OTA Toxicity in IPEC-J2 Cells

**Figure 7:**
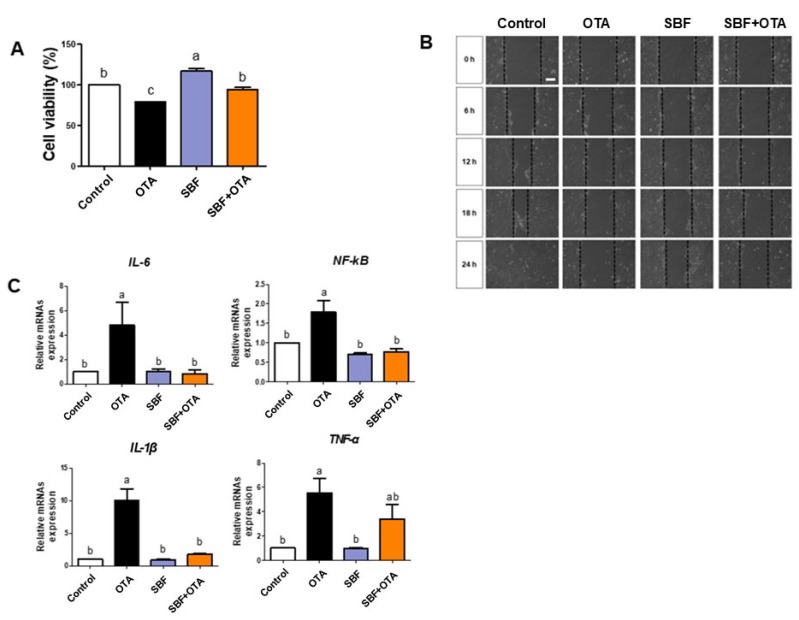
Spirobenzofuran mitigates the harmful effects of Ochratoxin A in IPEC-J2 cells. (**A**) Cell viability was measured in IPEC-J2 cells cotreated with OTA (5.5 μM) and Spirobenzofuran (10 ng/μL). (**B**) Effects of cell migration following OTA 5.5 μM, Spirobenzofuran 10 ng/μL, cotreatment (OTA 5.5 μM and Spirobenzofuran 10 ng/μL) at 0, 6, 12, 18, and 24 h. (**C**) The relative mRNA levels of inflammatory markers (*TNF-α*, *IL-1β*, *IL-6*, and *NF-kB*) following OTA 5.5 μM, Spirobenzofuran 10 ng/μL, cotreatment (OTA 5.5 μM and Spirobenzofuran 10 ng/μL) treatment compared with the control group. Lowercase letters (a, b, c) denote statistical differences between the control and the treatment groups. Error bars represent the standard errors (SEs) of three independent experiments.

We measured cell viability, cell migration, and the expression of inflammatory markers to determine whether Spirobenzofuran can regulate OTA toxicity in IPEC-J2 cells. The cell viability results exhibited that Spirobenzofuran, whether administered individually or in combination with OTA, significantly increased cell viability compared to OTA-treated cells (Figure 7A). Moreover, the wound width of cells treated with Spirobenzofuran individually or cotreated with OTA showed slight reductions compared to OTA-treated cells (Figure 7B). Finally, the mRNA expression of the inflammatory markers *TNF-α*, *IL-1β*, *IL-6*, and *NF-kB* was significantly decreased in cells treated with Spirobenzofuran, either individually or with OTA, compared to OTA-treated cells (Figure 7C).

## 4. Discussion

The intestinal epithelium is a vital organ in the livestock industry, as it not only facilitates nutrient transport but also acts as a barrier against deleterious substances in the host. Since the intestinal epithelium serves as the primary defense mechanism and maintains homeostasis in the host, it is vulnerable to foreign factors, such as pollutant forage, pathogens, and toxins [6,8]. When invaded by external factors, the immune system stimulates various signaling pathways, cytokine production, and leukocyte recruitment to eliminate these external factors and maintain homeostasis. However, continuous immune system activation can lead to reduced growth rates, malfunction, increased disease susceptibility, and chronic inflammatory responses [11,14]. Therefore, ensuring safe forage sanitation and maintaining an appropriate immune system are important functions of the intestinal epithelium in the livestock industry. OTA, a secondary metabolite produced by several molds, is a global issue due to its heat resistance and ability to flourish in hot and humid environments, leading to the contamination of livestock feed materials, such as corn, grains, cereals, and vegetables [3,32]. Increased OTA contamination can damage the intestinal epithelium, resulting in various side effects and economic losses [17,33,34]. Despite extensive research on the molecular mechanisms of OTA toxicity in the intestinal epithelium, further investigation and intervention for OTA-mediated issues are necessary.

We initially evaluated the effects of OTA exposure, such as cytotoxicity, inhibition of cell migration, and inflammatory responses, using porcine intestinal epithelium cell lines (IPEC-J2 cells) as an in vitro model. The OTA treatment conditions were analyzed using a WST-1 assay in a time-dependent manner with an IC50 value of 5.5 μM, as reported in a previous study. Our results showed decreasing trends of cell viability in a time-dependent manner with OTA treatment, with cell viability significantly decreased under 5.5 μM OTA treatment for 24 h. Consequently, treatment with 5.5 μM OTA for 24 h was used in subsequent experiments. Within the intestinal mucosa, damage caused by normal digestion, harmful factors, toxins, and inflammation is managed through a restoration process (proliferation, migration, and differentiation) among epithelial cells [35,36]. Numerous studies have reported that wound healing is modulated by various factors, including cell signaling, regulatory peptides, growth factors, cytokines, and AMPs [37,38,39]. We confirmed that OTA treatment inhibited cell migration in IPEC-J2 cells. Additionally, OTA treatment has been shown to induce inflammatory responses and upregulate inflammatory markers [31,40]. In this study, OTA treatment significantly increased the mRNA expression of inflammatory markers in IPEC-J2 cells, suggesting the harmful effects of OTA on IPEC-J2 cells.

AMPs, secreted from the epithelial surface of various species, have a relatively short amino acid structure and exhibit direct and indirect antimicrobial activity against various infectious agents. Apart from directly eliminating bacteria, AMPs exhibit immunomodulatory activity [41]. Thus, they serve multiple functions for intestinal health, including neutralizing endotoxins, recruiting immune cells, and enhancing the immune system and phagocytosis [19,42]. To date, numerous studies have demonstrated that AMPs have positive effects on intestinal epithelium when exposed to various infectious agents, including the inhibition of inflammatory markers, wound care, defense against external antigens, and maintenance of intestinal integrity [20,43,44]. Thus, we hypothesized that overexpressed AMPs may be a critical factor in protecting an intestinal epithelium exposed to mycotoxins. Among the AMPs, the beta-defensin family is mainly expressed by external factors, and overexpressed defensins have been shown to protect against several infectious agents in the porcine intestinal epithelium [45,46]. For DEFB1 overexpression in IPEC-J2 cells, we constructed a pcBD1 cell line through neomycin cell selection using a pcBD1 vector. Our results show that OTA decreased *DEFB1* mRNA expression in IPEC-J2 cells but conversely increased it in pcBD1 cells. Additionally, OTA-treated pcBD1 cells showed increased cell migration and a decrease in the mRNA expression of inflammatory markers (TNF-α, IL-1β, IL-6, and NF-kB) compared to OTA-treated IPEC-J2 cells.

Our findings support the hypothesis that overexpressed AMPs may be an important clue to protecting against mycotoxin exposure. For the OTA-mediated problem, we concluded that upregulation of DEFB1 was necessary and, therefore, conducted an HTS assay to discover candidate natural products that induce DEFB1. In this study, we screened 288 natural products, each in combination with OTA in IPEC-J2 cells, and found that 177 of these products exhibited higher cell viability than cells treated with OTA alone. Among these, we considered the 17 natural products with cell viability higher than 90% as potential candidates for mitigating OTA toxicity. Subsequently, we assessed the cell viability of randomly selected candidates ((2S)-4′,6-Dihydroxy-7-methoxyflavanone, 6,6′-Bieckol, Spirobenzofuran, and 4′-Hydroxydehydrokawain) at various concentrations to verify their cytotoxicity and determine their optimal concentrations. Compared to the control, there was no statistical difference in the cell viability of 6,6′-bieckol, Spirobenzofuran, and 4′-Hydroxydehydrocawain 20 ng/μL, with their cell viability at 10 ng/μL showing a significant increase. The cell viability of (2S)-4′,6-Dihydroxy-7-methoxyflavanone at 20 ng/μL slightly decreased, and at 10 ng/μL, there were no significant differences compared to the control. These data confirmed the cytotoxicity of the candidates and determined 10 ng/μL as the appropriate concentration for further experiments.

To classify DEFB1-inducing candidates, we confirmed the DEFB1 expression of (2S)-4′,6-Dihydroxy-7-methoxyflavanone, 6,6′-Bieckol, Spirobenzofuran, and 4′-Hydroxydehydrokawain in IPEC-J2 cells with or without OTA treatment. We ascertained that DEFB1 expression of individual (2S)-4′,6-Dihydroxy-7-methoxyflavanone, 6,6′-Bieckol, 4′-Hydroxydehydrokawain or them combined with OTA treatment abnormal increased and decreased trend. However, Spirobenzofuran, when administered individually or combined with OTA, revealed an increased DEFB1 expression. We thus noted and selected Spirobenzofuran as a potential natural product capable of modulating OTA exposure.

Spirobenzofuran is a bioactive metabolite obtained from mushrooms of the genus *Acremonium* and *Coprinus* [47]. Previous studies have reported the antioxidant, anti-inflammatory, antifungal, and antipathogenic effects of these genera [48,49,50]. Although the positive effects of these genera have been investigated, studies on Spirobenzofuran derived from these genera are scarce. We found that Spirobenzofuran administration alleviated OTA-induced cytotoxicity and inhibited cell migration in IPEC-J2 cells. Furthermore, we confirmed that Spirobenzofuran administration did not induce inflammatory markers, and cotreatment with OTA slightly decreased the expression of IL-6, NF-kB, and IL-1β and TNF-α compared to OTA-induced IPEC-J2 cells.

## 5. Conclusions

**Figure 8:**
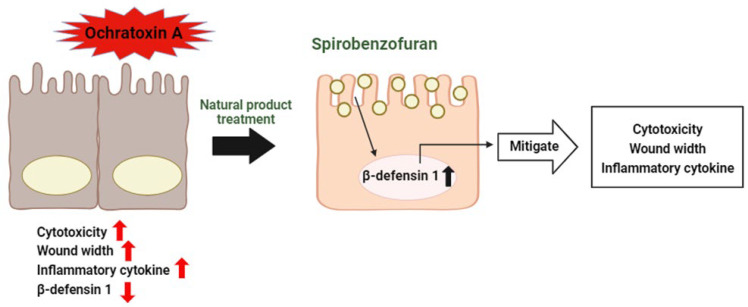
Schematic illustrating the current hypothesis regarding the regulation of DEFB1 in OTA-exposed intestinal epithelial cells. OTA affects cytotoxicity, wound repair, and the production of inflammatory markers, and OTA-mediated adverse effects are alleviated through an increase in DEFB1.

Our study showed that OTA treatment has adverse effects, such as cytotoxicity, decreased cell migration, and the induction of inflammatory markers in porcine intestinal epithelial cells. However, we found that OTA-induced inhibition of cell migration and the induction of inflammatory markers were modulated in pcBD1 cells, overexpressed DEFB1 cell lines (Figure 8). Based on these findings, Spriobenzofuran, a natural product selected through the HTS assay and identified for its DEFB1-inducing properties, was revealed to mitigate the harmful effects of OTA-exposed intestinal epithelium. Collectively, these data could be indicated to provide clues about porcine intestinal epithelium threatened by mycotoxin. Moreover, our study may help us understand and select candidate feed additives for the pig industry. 

## Figures and Tables

**Table 1:** List of primers.

Genes	Description	Accession No.		Sequence (5′–3′)
*GAPDH*	*Glyceraldehyde-3-phosphate dehydrogenase*	*NM_001206359*	Forward	ACACCGAGCATCTCCTGACT
			Reverse	GACGAGGCAGGTCTCCCTAA
*TNF-α*	*Tumor necrosis factor α*	*NM_214022*	Forward	TTTCTGTGAAAACGGAGCTG
			Reverse	CAGCGATGTAGCGACAAGTT
*IL-1β*	*Interleukin-1β*	*NM_001305893*	Forward	GAACAAGAGCATCAGGCAGA
			Reverse	TGGCATCACAGACAAAGTCA
*IL-6*	*Interleukin-6*	*NM_001252429*	Forward	GCTTCCAATCTGGGTTCAAT
			Reverse	ATTCTTTCCCTTTTGCCTCA
*NF-kB*	*Nuclear factor of kappa light polypeptide gene enhancer in B-cells 1*	*NM_001048232*	Forward	TCTCTGACGGTGAAGTGTCC
			Reverse	AAGTTGGCATTTTTGGAAGG
*DEFB1*	*Beta-defensin 1*	*NM_213838*	Forward	TCCTCCTTGTATTCCTCCTCA
			Reverse	TCTGTGGGGTTGTTTCTTCA

## Data Availability

The original contributions presented in the study are included in the article/Supplementary Material, further inquiries can be directed to the corresponding author.

## Supplementary Materials

The following supporting information can be downloaded at: https://www.mdpi.com/article/10.3390/toxics12070487/s1, Figure S1: DEFB1 mRNA expression of candidate natural products and alteration upon OTA exposure.
