# Supplementary figures and images for: Spirobenzofuran Mitigates Ochratoxin A-Mediated Intestinal Adverse Effects in Pigs through Regulation of Beta Defensin 1
Source: Toxics. 2024 Jul 3;12(7):487. doi: 10.3390/toxics12070487 (PMC11281199; doi:10.3390/toxics12070487)

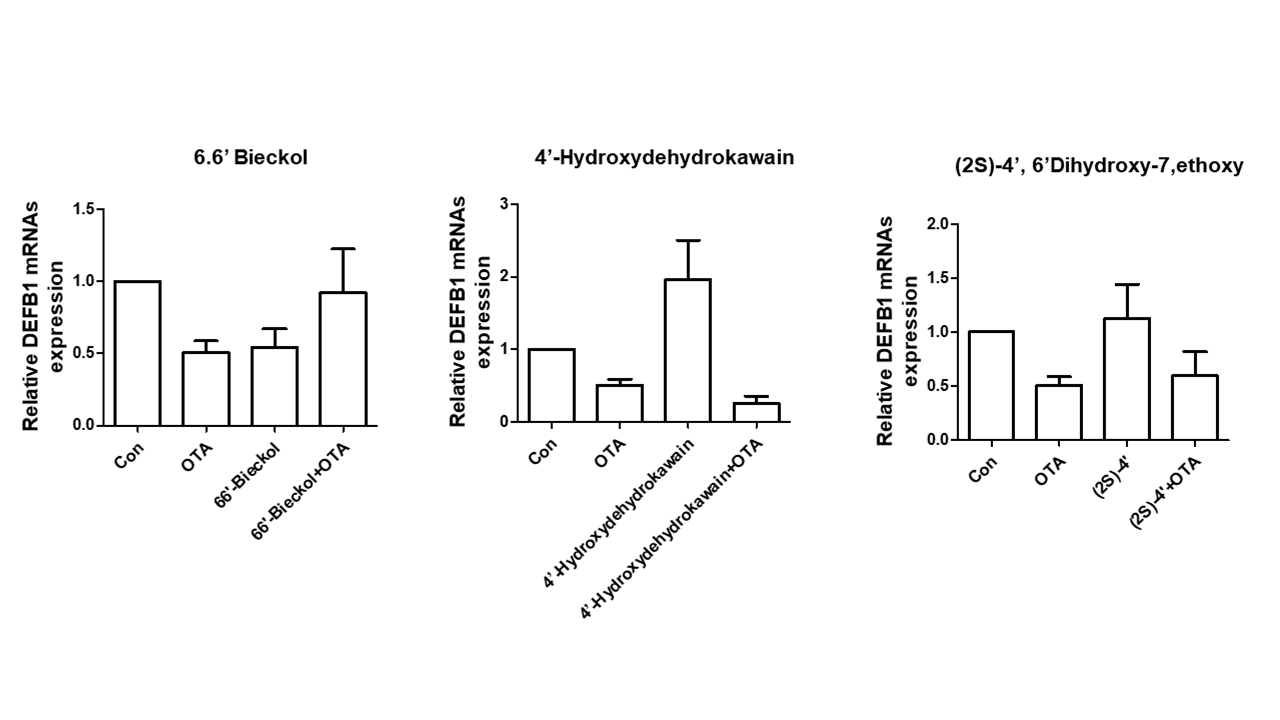

Supplement: Supplementary file 1 [file toxics-12-00487-s001.zip › toxics-3082250-supplementary.tif]
